# Synthesis, Characterization and Biological Properties of Tridentate NNO, NNS and NNN Donor Thiazole-Derived Furanyl, Thiophenyl and Pyrrolyl Schiff Bases and Their Co(II), Cu(II), Ni(II) and Zn(II) Metal Chelates

**DOI:** 10.1155/MBD.2000.17

**Published:** 2000

**Authors:** Zahid H. Chohan, Samina Kausar

**Affiliations:** Department of Chemistry Islamia University Bahawalpur Pakistan

## Abstract

2-Aminothiazole undergoes condensation reactions with furane-, thiophene- and pyrrole-2-carboxylaldehyde to give tridentate NNO, NNS and NNN Schiff bases respectively. These tridentate Schiff bases formed complexes of the type [M (L)_2_]X_2_ where [M=Co(II), Cu(II), Ni(II) or Zn(II), L=N-(2-furanylmethylene)-2-aminothiazole (L^1^), N-(2-thiophenylmethylene)-2-aminothiazole (L^2^, N-(2-pyrrolylmethylene)-2-aminothiazole (L^3^) and X=Cl. The structures of these Schiff bases and of their complexes have been determined on the basis of their physical, analytical and spectral data. The screening results of these compounds indicated them to possess excellent antibacterial activity against tested pathogenic bacterial organisms e.g., *Escherichia coli*, *Staphylococcus aureous* and *Pseudomonas aeruginosa*. However, in comparison, their metal chelates have been shown to possess more antibacterial activity than the uncomplexed Schiff bases.


### SYNTHESIS, CHARACTERIZATION AND BIOLOGICAL PROPERTIES OF TRIDENTATE NNO, NNS AND NNN DONOR THIAZOLE-DERIVED FURANYL, THIOPHENYL AND PYRROLYL SCHIFF BASES AND THEIR Co(II), Cu(II), Ni(II) AND Zn(II) METAL CHELATES

Zahid H. Chohan* and Samina Kausar

Department of Chemistry, Islamia University, Bahawalpur, Pakistan
Email <dr.zahid@bwp.nexlinx.net.pk>

**ABSTRACT**
2-Aminothiazole undergoes condensation reactions with furane-, thiophene- and pyrrole-2-carboxylaldehyde to give tridentate NNO, NNS and NNN Schiff bases respectively. These tridentate Schiff bases formed complexes of the type $[M(L)_2]X_2$ where [M= Co(II), Cu(II), Ni(II) or Zn(II), L= N-(2-furanylmethylene)-2-aminothiazole ($L^1$), N-(2-thiophenylmethylene)-2-aminothiazole ($L^2$), N-(2-pyrrolylmethylene)-2-aminothiazole ($L^3$) and X= Cl. The structures of these Schiff bases and of their complexes have been determined on the basis of their physical, analytical and spectral data. The screening results of these compounds indicated them to possess excellent antibacterial activity against tested pathogenic bacterial organisms e.g., *Escherichia coli, Staphylococcus aureous* and *Pseudomonas aeruginosa.* However, in comparison, their metal chelates have been shown to possess more antibacterial activity than the uncomplexed Schiff bases.

### INTRODUCTION

Thiazole and related compounds are of considerable interest[1-4] due to their presense in the histidyl residue of proteins. As a ligand it also provides a potential binding site for metal ions. A thorough knowledge is therefore required to understand their coordination properties and the role of metal ions in such systems. A limited amount of work has been published on the complexing properties of such related compounds. This work is an extension of previously reported[5-7] studies on the coordination chemistry and the role of metal ions on such compounds and their bioability as bactericidals. Sherman and Dickson have reported[8] series of 2-amino-4-(5-nitro-2-furyl)thiazoles and their various chloro, hydroxy and methoxy derivatives and observed that these compounds exhibit antibacterial activity in vitro, particularly against *Staphylococcus aureus, Salmonella* and *Escherichia coli.* Subsequently a number of reports[9,10] and patents[11] have described the preparation and antibacterial properties of a variety of hydroxyaryliminomethylthiazole, hydroxynaphthylthiazole-2-yl-thiazolidone and α-substituted benzylaminothiazoles and their derivatives. However, no work so far, is reported on the synthesis and antibacterial properties of furane-, thiophene- and pyrrole-derived Schiff bases of thiazole. We therefore have made an effort to present, in this paper, the synthesis and biological activity of hitherto some novel Schiff bases ($L^1$, $L^2$ and $L^3$) containing the thiazole nucleus coupled with furane, thiophene and pyrrole moieties (Figure 1).

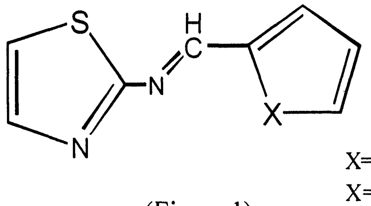

X=O; $L^1$
X=S; $L^2$
X=NH; $L^3$

(Figure 1)

The Co(II), Cu(II), Ni(II) and Zn(II) metal chelates of the title thiazole-derived compounds have been synthesised and characterized by their physical, analytical and spectral data. In order to evaluate their antibacterial properties the synthesised Schiff bases and their complexes have been screened against bacterial species *Escherichia coli, Staphylococcus aureous, Pseudomonas aeruginosa* and *Klebsiella pneumonae.* The results of these studies have indicated that all these Schiff bases are antibacterial against one or more bacterial species and that, upon complexation, their antibacterial properties become more pronounced.

### EXPERIMENTAL

### Material and Methods

All chemicals and solvents used were of Analar grade. IR and NMR spectra were recorded on Philips Analytical PU 9800 FTIR spectrophotometer and on a Brucker 250 MHz spectrometer. UV- Visible spectra were obtained on a Hitachi U-2000 double-beam spectrophotometer. Conductance of the metal complexes was determined in DMF on a YSI-32 model conductometer. Magnetic measurements were done on solid complexes using the Gouy method. Melting points were recorded on a Gallenkamp apparatus and are uncorrected.

### Preparation of the Ligands

**N-(2-Furanylmethylene)-2-aminothiazole ($L^1$)**

An amount of furane-2-aldehyde (0.83 mL, 0.97 g, 0.01 M) in absolute ethanol (20 mL) was added to a stirred ethanolic solution (20 mL) of 2-aminothiazole (1 0 g,.0.01 M). Then 2-3 drops of conc. $H_2SO_4$ were added in it and the mixture,refluxed for 1 h. The reaction mixture was then cooled and left for 24 h at room temperature. During this period yellow needles were formed. The crystals thus formed were filtered, washed with ethanol and dried to give $L^1$ (85 g, 52%), m.p 158°C.

The same method was applied for the preparation of Schiff bases $L^2$ (1.02 g, 55%) and $L^3$ (0.95 g, 56%) by using their respective aldehydes, thiophene-2-carboxaldehyde and pyrrole-2-aldehyde.

### Preparation of Metal Complexes

An ethanol solution (20 mL) of the appropriate metal(II) salt (0.001 M) was added to a stirred ethanol solution (15 mL) of the respective Schiff base (0.002 M). The mixture was refluxed for 2 h. The resulting mixture was cooled, filtered and reduced to nearly half its volume. The concentrated solution was left overnight at room temperature which resulted in the formation of a solid product. The product thus obtained was filtered, washed with ethanol, then with ether and dried. Crystallization from aqueous ethanol gave the desired metal complexes **1** (65 %), **2** (60 %), **3** (62 %), **4** (65 %), **5** (60 %), **6** (62 %), **7** (65 %), **8** (65 %), **9** (62 %), **10** (63 %), **11** (60 %) and **12** (65 %).

### Antibacterial Studies

The synthesized metal chelates and, for comparison purposes, the free Schiff bases were screened for their antibacterial activity against pathogenic bacterial species, *Escherichia coli, Staphylococcus aureus* and *Pseudomonas aeruginosa* and *Klebsiella pneumonae* The paper disc diffusion method was adopted for the determination of the antibacterial activity as reported[12] elsewhere.

### RESULTS AND DISCUSSION

### Physical Properties

Schiff bases were prepared by reacting equimolar amounts of the respective furane-2-, thiophene-2- and pyrrole-2-aldehydes with 2-aminothiazole in ethanol. The structures of these ligands were established with the help of their IR, $^1$H NMR, $^{13}$C NMR and microanalytical data (Tables 1 and 4)

All the metal complexes (1-12) (Table 2) of these Schiff bases were prepared by the stoichiometric reaction of the respective metals as their chlorides and ligands in molar ratio M:L = 1:2. Their elemental analyses and molecular weights shows the monomeric nature of these complexes. All the synthesized complexes are air stable, non-hygroscopic solids and decompose without melting. They are all soluble in DMF and DMSO. Low conductance values of the complexes in DMF solution (12-28 ohm$^{-1}$cm$^2$mol$^{-1}$) indicated[13] that they are all non-electrolytic in nature

Table 1. Physical, Spectral and Analytical Data of the Schiff bases

| Ligand | M.P (°C) | IR (cm$^{-1}$) | Calc (Found) % C | H | N |
|---|---|---|---|---|---|
| $L^1$ $C_8H_6N_2OS$ [178.0] | 135 | 3210, 2925, 2024, 1635, 1515, 1540, 1370, 1115, 1060, 950 | 53.9 (54.2) | 3.4 (3.3) | 15.7 (15.9) |
| $L^2$ $C_8H_6N_2S_2$ [198.1] | 148 | 3215, 2915, 2024, 1635, 1515, 1540, 1375, 1110, 1065, 950 | 48.5 (48.4) | 3.0 (3.4) | 14.1 (14.3) |
| $L^3$ $C_8H_7N_3S$ [177.1] | 125 | 3215, 2930, 2024, 1635, 1515, 1540, 1372, 1112, 1065, 950 | 54.2 (54.6) | 4.0 (3.8) | 23.7 (23.9) |

### Infrared spectra

IR spectra of the Schiff bases showed the absence of bands at 1735 and 3420 cm$^{-1}$ due to carbonyl ν (C=O) and ν ($NH_2$) stretching vibrations and, instead, the appearance of a strong new band at ~ 1635 cm$^{-1}$ assigned[14-16] to the azomethine ν (C=N) linkage. This suggested that the amino and aldehyde moieties of the starting reagents no more exist and have been converted into the respective Schiff base linkages. The comparison of the infrared spectra of the ligands and their metal chelates indicated that the ligands were coordinated to the metal atom in three ways, thus representing the ligands to act as tridentates. The bands appearing at 1635 and 1615 $cm^{-1}$ assigned to azomethine and thiazole ring ν (C=N) vibrations shifted to lower frequency by 5-10 $cm^{-1}$ indicating the participation of the azomethine and thiazole ring nitrogens in chelation. Further conclusive evidence of the co-ordination of these tridentate ligands with the metals was shown by the appearance of weak low frequency new bands at ~ 360-365, ~ 455-460 and ~ 525-530 $cm^{-1}$ (Table 2). These were in turn assigned to metal-sulphur ν (M-S) (in thienyl compounds), metal-oxygen ν (M-O) (in furanyl derivatives) and metal-nitrogen ν (M-N) (in the pyrrolyl compounds) stretching vibrations[15] respectively. These new bands were observable only in the spectra of the metal complexes and not in the spectra of their Schiff bases thus confirming the participation of the heteroatoms S, O and N to the coordination.

Table 2. Physical and Analytical Data of the Metal(II) Chelates

| No | Metal chelate/ Mol. Formula | M.P($^oC$) (dec) | B.M. ($\mu_{eff}$) | Cal (Found)% C H N |
|---|---|---|---|---|
| 1 | $[Co(L^1)_2]Cl_2$ [486.0] $C_{16}H_{12}CoCl_2N_4O_2S_2$ | 221-223 | 4.9 | 39.5 2.5 11.5 (39.8) (2.5) (11.1) |
| 2 | $[Co(L^2)_2]Cl_2$ [526.1] $C_{16}H_{12}CoCl_2N_4S_4$ | 234-236 | 4.7 | 36.5 2.3 10.6 (36.7) (2.6) (10.4) |
| 3 | $[Co(L^3)_2]Cl_2$ [484.0] $C_{16}H_{14}CoCl_2N_6S_2$ | 251-253 | 4.8 | 39.7 2.9 17.4 (40.0) (2.8) (17.1) |
| 4 | $[Cu(L^1)_2]Cl_2$ [490.6] $C_{16}H_{12}CuCl_2N_4O_2S_2$ | 257-260 | 1.7 | 39.1 2.4 11.4 (39.3) (2.6) (11.3) |
| 5 | $[Cu(L^2)_2]Cl_2$ [530.7] $C_{16}H_{12}CuCl_2N_4S_4$ | 242-244 | 1.7 | 36.2 2.3 10.6 (36.0) (2.5) (10.4) |
| 6 | $[Cu(L^3)_2]Cl_2$ [488.6] $C_{16}H_{14}CuCl_2N_6S_2$ | 232-234 | 1.8 | 39.3 2.9 17.2 (39.7) (2.7) (17.1) |
| 7 | $[Ni(L^1)_2]Cl_2$ [485.7] $C_{16}H_{12}NiCl_2N_4O_2S_2$ | 212-214 | 3.1 | 39.5 2.5 11.5 (39.6) (2.6) (11.4) |
| 8 | $[Ni(L^2)_2]Cl_2$ [525.8] $C_{16}H_{12}NiCl_2N_4S_4$ | 226-228 | 3.3 | 36.5 2.3 10.7 (36.7) (2.0) (10.9) |
| 9 | $[Ni(L^3)_2]Cl_2$[483.7] $C_{16}H_{14}NiCl_2N_6S_2$ | 243-245 | 3.3 | 39.0 2.9 17.4 (39.4) (2.8) (17.1) |
| 10 | $[Zn(L^1)_2]Cl_2$ [492.4] $C_{16}H_{12}ZnCl_2N_4O_2S_2$ | 233-235 | Dia | 39.0 2.4 11.4 (39.2) (2.7) (11.0) |
| 11 | $[Zn(L^2)_2]Cl_2$ [532.5] $C_{16}H_{12}ZnCl_2N_4S_4$ | 252-254 | Dia | 36.0 2.3 10.5 (36.4) (2.4) (10.7) |
| 12 | $[Zn(L^3)_2]Cl_2$ [490.4] $C_{16}H_{14}ZnCl_2N_6S_2$ | 257-259 | Dia | 39.2 2.9 17.1 (39.5) (3.1) (17.3) |

Table 3. IR and UV-Visible Spectral Data of the Metal(II) Chelates

| Complex | IR ($cm^{-1}$) | $\lambda_{max}$ ($cm^{-1}$) |
|---|---|---|
| 1 | 1630 (HC=N), 1605 (C=N), 455 (M-O), 525 (M-N) | 30115, 18625, 7875 |
| 2 | 1625 (HC=N), 1610 (C=N), 460 (M-O), 525 (M-N) | 30775, 17555, 8915 |
| 3 | 1625 (HC=N), 1610 (C=N), 460 (M-O), 530 (M-N) | 30545, 17950, 8430 |
| 4 | 1630 (HC=N), 1605 (C=N), 455 (M-O), 525 (M-N) | 28635, 22345, 16220 |
| 5 | 1630 (HC=N), 1610 (C=N), 360 (M-S), 525 (M-N) | 27555, 23670, 17635 |
| 6 | 1630 (HC=N), 1605 (C=N), 365 (M-S), 530 (M-N) | 28220, 22875, 16770 |
| 7 | 1625 (HC=N), 1605 (C=N), 365 (M-S), 525 (M-N) | 28525, 15545, 10110 |
| 8 | 1630 (HC=N), 1605 (C=N), 365 (M-S), 530 (M-N) | 29200, 16270, 9415 |
| 9 | 1635 (HC=N), 1610 (C=N), 535 (M-N) | 28675, 15855, 9615 |
| 10 | 1630 (HC=N), 1610 (C=N), 525 (M-N) | 28250, 13875 |
| 11 | 1625 (HC=N), 1605 (C=N), 530 (M-N) | 28845, 13325 |
| 12 | 1630 (HC=N), 1605 (C=N), 525 (M-N) | 28435, 13565 |

**NMR Spectra**

The $^1H$ NMR and $^{13}C$ NMR spectra of the free ligand (Table 4) and its metal complexes support the conclusions derived from the IR spectra. The $^1H$ NMR spectra of the free ligands exhibited[17] peaks at δ 7.8 ppm due to azomethine proton (CH=N). Other heteroaromatic protons were also found in their expected region. The azomethine proton signal in the spectra of the complexes display an upfield shift indicating in turn, its involvement in coordination. A thiazole proton of the free ligand at δ 7.4 ppm also showed a downfield shift in the spectra of complexes providing an evidence for the coordination of the thiazole nitrogen to the metal atom. Similarly, $^{13}C$ NMR spectra of the ligands showed azomethine carbon resonance at δ 165.4 ppm and one of the thiazole carbon resonance at δ 143.3 ppm which shifted downfield in the complexes attributed[18] to the coordination of azomethine and thiazole nitrogens to the metal atom. Similar shifts were observed in the resonance of furyl-, thiophenyl and pyrrolyl moities suggesting the coordination of their heteroatoms to the corresponding metal atom.

Table 4. NMR Data of the Schiff bases

| $L^n$ | $^1H$ NMR | $^{13}C$ NMR |
|---|---|---|
| $L^1$ | 4.5-4.6 (m, 1H, furanyl), 4.8 (dd, 1H, furanyl), 5.7 (s, 1H, furanyl), 6.2 (s, 1H, azomethine), 7.4 (d, 1H, thiazole), 7.8 (d, 1H, thiazole) | 118.7, 143.4, 155.8 (thiazole), 162.4 (azomethine), 106.3, 112.7, 121.4, 124.1 (furanyl) |
| $L^2$ | 4.6-4.7 (m, 1H, thionyl), 4.8 (dd, 1H, thionyl), 5.6 (s, 1H, thionyl), 6.3 (s, 1H, azomethine), 7.4 (d, 1H, thiazole), 7.8 (d, 1H, thiazole) | 118.3, 143.2, 155.6 (thiazole), 162.8 (azomethine), 106.2, 112.5, 121.2, 124.1 (thionyl) |
| $L^3$ | 4.6-4.7 (m, 1H, pyrrolyl), 4.8 (dd, 1H, pyrrolyl), 5.7(s, 1H, pyrrolyl), 6.3 (s, 1H, azomethine), 7.3 (d, 1H, thiazole), 7.9 (d, 1H, thiazole), 8.5 (s, 1H,NH). | 118.9, 143.6, 155.8 (thiazole), 162.6 (azomethine), 107.1, 112.8, 121.7, 124.5 (pyrrolyl) |

**Magnetic Moments and UV-Visible Spectra**

The room temperature magnetic moment of the solid cobalt(II) complexes was found to be 4.8-4.9 B.M, indicative[19] of three unpaired electrons per Co(II) ion in an octahedral environment. The copper(II) complexes displayed $\mu_{eff}$ values, 1.7-1.8 B.M showing the presence of one unpaired electron per Cu(II) ion suggesting a distorted octahedral geometry. The nickel(II) complexes also showed $\mu_{eff}$ values in the range 3.1-3.2 B.M indicative[20] of two unpaired electrons per Ni(II) ion for their ideal octahedral configuration.

The electronic spectra of the the Co(II) chelates showed three bands observed at 7875-8915 $cm^{-1}$, 17555-18625 $cm^{-1}$ and 30115-30775 $cm^{-1}$ which may be assigned to $^4T_{1g} \rightarrow ^4T_{2g}$ (F), $^4T_{1g} \rightarrow ^3A_{2g}$(F) and $^4T_{1g} \rightarrow ^4T_{1g}$ (P) transitions respectively and are suggestive[21,22] of an octahedral geometry around the cobalt ions. The electronic spectra of the Cu(II) complexes similarly showed three bands in the regions 16220-17635 $cm^{-1}$ , 22345-23670 $cm^{-1}$ and 27555-28635 $cm^{-1}$. The low energy band may be assigned for Cu(II) in an octahedral configuration[23] corresponding to the transitions $^2E_g \rightarrow ^2T_{2g}$ and to the symmetry forbidden ligand → metal charge transfer. The Ni(II) complexes exhibited three spin-allowed bands at 9415-10110 $cm^{-1}$, 15545-16270 $cm^{-1}$ and 28525-29200 $cm^{-1}$ assignable[24] respectively, to the transitions $^3A_{2g}$ (F)$\rightarrow ^3T_{2g}$ (F)($\nu_1$), $^3A_{2g}$(F)$\rightarrow ^3T_{1g}$(F) )($\nu_2$) and $^3A_{2g}$(F)$\rightarrow ^3T_{2g}$(P) ($\nu_3$) which are suggestive of their octahedral geometry. The electronic spectra of the Zn(II) complexes exhibited a high intensity band at 28250-28845 $cm^{-1}$ assigned to ligand-metal charge-transfer and a band at 13325-13875 $cm^{-1}$ due to transitions $^2E_g \rightarrow ^2T_{2g}$ in a distorted octahedral environment[25] (Fig 2).

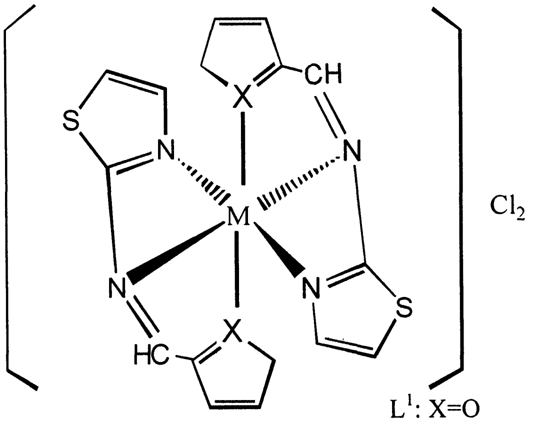

$L^1$: X=O
$L^2$: X=S
M=Co(II), Cu(II), Ni(II) & Zn(II)

(Figure 2) Proposed Structure for the Metal Chelates

**Antibacterial Properties**

The title ligands and their metal chelates were evaluated for their antibacterial activity against the bacterial species *Escherichia coli (a), Pseudomonas aeruginosa (b), Staphylococcus aureus (c)* and *Klebsiella pneumonae (d).* The compounds were tested at a concentration of 30 μg/0.01 mL in DMF solution using the paper disc diffusion method. The susceptibility zones were measured in mm and reproduced in Table 5. The susceptibility zones were the clear zones around the discs. All the Schiff bases were found to be biologically active and their metal complexes showed more significant antibacterial activities against one or more bacterial species in comparison to the uncomplexed Schiff bases. It is however definite that in most of the cases chelation tends to make the ligands act as more powerful and potent bactericidals, thus killing more of the bacteria than the parent Schiff bases. It is however suspected that factors such as solubility, conductivity, dipole moment and cell permeability mechanisms (influenced by the presence of metal ions) may be possible reasons for increasing this activity.

Table 5. Antibacterial Activity Data

| Schiff base/ Chelate | Microbial Species a | b | c | d |
|---|---|---|---|---|
| $L^1$ | ++ | + | + | ++ |
| $L^2$ | ++ | + | ++ | + |
| $L^3$ | ++ | + | + | ++ |
| **1** | +++ | ++ | +++ | +++ |
| **2** | +++ | +++ | ++ | ++ |
| **3** | +++ | ++ | +++ | ++++ |
| **4** | +++ | ++ | ++ | +++ |
| **5** | ++++ | +++ | +++ | +++ |
| **6** | +++ | +++ | +++ | ++ |
| **7** | ++++ | ++ | +++ | +++ |
| **8** | ++ | +++ | + | ++++ |
| **9** | +++ | +++ | +++ | ++ |
| **10** | +++ | +++ | +++ | +++ |
| **11** | +++ | +++ | ++ | +++ |
| **12** | ++ | +++ | +++ | ++ |

a= *Escherichia coli,* b= *Staphylococcus aureus,*
c= *Pseudomonas aeruginosa* d= *Klebsiella pneumonae*
Inhibition zone diameter mm (% inhibition): +, 6-10 (27-45 %); ++, 10-14 (45-64 %); +++, 14-18 (64-82 %); ++++, 18-22 (82-100 %). Percent inhibition values are relative to inhibition zone (22 mm) of the most active compound with 100 % inhibition.

### ACKNOWLEDGEMENT

The author gratefully acknowledges the help of Department of Pathology, Qaid-e-Azam Medical College, Bahawalpur, in undertaking the antibacterial studies.

### REFERENCES

1. J. V. Metzger in "Comprehensive Heterocyclic Chemistry", Eds, A. R. Katritzky and C. W. Rees, Pergman, New York, Vol 6, 1984.
2. M. Patra, S. K. Mahapatra and B. Dash, *J. Ind. Chem. Soc*, **51**, 1031 (1974).
3. S. C. Sharma, *Bull. Chem. Soc. Jpn*, **40**, 2422 (1967).
4. D. K. Johnson, T. B. Murphy, T. B. Rose, W. H. Goodwin and L. Pickart, *Inorg. Chim. Acta*, **67**, 159 (1982).
5. W. R. Sherman and D. E. Dickson, *J. Org. Chem*, **27**, 1351 (1962).
6. T. Akira and U. Toshinao, *Chem. Pharm. Bull*, **26**, 3576 9191978).
7. B. S. Holla, B. Kalurraya and K. R. Sridhar, *Curr. Sci*, **55**, 73 (1986).
8. D. Modi, S. S. Sabnis and C. B. Deliwala, *J. Med. Chem*, **13**, 935 (1970).
9. G. Contneras and H. Cortes, *Inorg. Nucl. Chem Lett*, **7**, 639 (1970).
10. T. Ujhe, *Ann. Rept. Canc. Inst,. Kanazawa. Jpn*, **1**, 109 (1967).
11. R. J. Alaimo, *US Pat.* 4, 012, 409 (Cl 260-305, COD7 419/00), 1977, *Chem Abstr*, **87**, 5952 (1977).
12. Z. H. Chohan and A. Rauf, *Synth. React. Inorg. Met-Org. Chem*, **26**, 592 (1996).
13. A. M. Shallary, M. M. Moustafa and M. M. Bekheit, *J. Inorg. Nucl. Chem*, **41**, 267 (1979).
14. J. E. Kovacic, *Spectrochim. Acta*, 23A, 183 (1967).
15. D. M. Adams, "Metal Ligands and Related Vibrations", Edward Arnold, London, 1967.

16. K. Nakamoto, "Infrared Spectra of Inorganic and Coordination Compounds", John Wiley, New York, 1963.
17. T. J. Batterham, "NMR Spectra of Simple Heterocycles", Wiley Interscience, New York, 1973.
18. D. H. Williams and I. Fleming, "Spectroscopic Methods in Organic Chemistry", 4$^{th}$ Ed, Mac Graw Hill, London, 1989.
19. M. D. Glick and R. L. Lintvedt, *Prog. Inorg. Chem*, **21**, 233 (1976),
20. E. K. Barefield, D. H. Busch and S. M. Nelson, *Quart. Rev*, **22**, 457 (1968).
21. B. N. Figgis, "Introduction to Ligand Fields", J. Wiley, New York (1976).
22. B. P. Lever, "Inorganic Electronic Spectroscopy", Elsevier, Amsterdam (1984).
23. C. J. Balhausen, "Introduction to Ligand Fields", McGraw Hill, New York (1962).
24. D. W. Meek, R. S. Drago and T. S. Piper, *Inorg. Chem.*, **1**, 285 (1962).
25. A. D. Liehr, *J. Phys. Chem.*, **67**, 1314, (1967).

**Received: October 29, 1999 - Accepted: November 24, 1999 -**
**Received in revised camera-ready format: November 25, 1999**

